# AHR Over-Expression in Papillary Thyroid Carcinoma: Clinical and Molecular Assessments in a Series of Italian Acromegalic Patients with a Long-Term Follow-Up

**DOI:** 10.1371/journal.pone.0101560

**Published:** 2014-07-14

**Authors:** Caterina Mian, Filippo Ceccato, Susi Barollo, Sara Watutantrige-Fernando, Nora Albiger, Daniela Regazzo, Paola de Lazzari, Gianmaria Pennelli, Sandra Rotondi, Davide Nacamulli, Maria Rosa Pelizzo, Marie-Lise Jaffrain-Rea, Franco Grimaldi, Gianluca Occhi, Carla Scaroni

**Affiliations:** 1 Endocrinology Unit, Department of Medicine, University-Hospital of Padua, Padua, Italy; 2 II Pathology Unit, Department of Medicine, University-Hospital of Padua, Padua, Italy; 3 Department of Clinical and Biotechnological Sciences, University of L'Aquila, L'Aquila, Italy; 4 Surgical Pathology Unit, Department of Surgery, University-Hospital of Padua, Padua, Italy; 5 Neuromed Institute, Department of Neurological Sciences, University of L'Aquila, L'Aquila, Italy; 6 Endocrinology and Metabolism Unit, University-Hospital “Santa Maria della Misericordia,” Udine, Italy; IPATIMUP/Faculty of Medicine of the University of Porto, Portugal

## Abstract

**Aim:**

Acromegaly reportedly carries an increased risk of malignant and benign thyroid tumors, with a prevalence of thyroid cancer of around 3–7%. Germline mutations in the aryl-hydrocarbon receptor (AHR) interacting protein (AIP) have been identified in familial forms of acromegaly. The molecular and endocrine relationships between follicular thyroid growth and GH-secreting pituitary adenoma have yet to be fully established. Our aim was to study the prevalence of differentiated thyroid cancer (DTC) in acromegaly, focusing on the role of genetic events responsible for the onset of thyroid cancer.

**Methods:**

Germline mutations in the *AIP* gene were assessed in all patients; *BRAF* and *H-N-K RAS* status was analyzed by direct sequencing in thyroid specimens, while immunohistochemistry was used to analyze the protein expression of AIP and AHR. A set of PTCs unrelated to acromegaly was also studied.

**Results:**

12 DTCs (10 papillary and 2 follicular carcinomas) were identified in a cohort of 113 acromegalic patients. No differences in GH/IGF-1 levels or disease activity emerged between patients with and without DTC, but the former were older and more often female. *BRAF* V600E was found in 70% of the papillary thyroid cancers; there were no *RAS* mutations. AIP protein expression was similar in neoplastic and normal cells, while AHR protein was expressed more in PTCs carrying BRAF mutations than in normal tissue, irrespective of acromegaly status.

**Conclusions:**

The prevalence of DTC in acromegaly is around 11% and endocrinologists should bear this in mind, especially when examining elderly female patients with uninodular goiter. The DTC risk does not seem to correlate with GH/IGF-1 levels, while it may be associated with *BRAF* mutations and AHR over-expression. Genetic or epigenetic events probably play a part in promoting thyroid carcinoma.

## Introduction

Acromegaly is an endocrine disorder due mainly to GH-secreting pituitary adenoma [1], which carries an increased risk of mortality due to cardiovascular and respiratory complications [2]. Many authors have also suggested that acromegaly is associated with a higher likelihood of benign and malignant neoplasms [2]–[4], leading to a mortality rate of around 25% for cancer-related complications [5], [6]. Both *in vitro* and *in vivo* studies clearly support a role for the GH/IGF-1 axis in tumor development as a consequence of its mitogenic and anti-apoptotic actions in many tissues [6]. Some authors also claim that the involvement of other pro-oncogenic factors such as insulin [7], or genetic and epigenetic influences predisposing to GH-secreting tumors [8], might also promote the onset of various neoplasms.

Acromegaly have a high prevalence of goiter and differentiated thyroid carcinoma (DTC) [9]–[17], the latter ranging from 5.6% to 10.6% [9]–[17]. This means that thyroid gland morphology should be assessed regularly in these patients.

Papillary thyroid carcinoma (PTC) is the most frequently reported thyroid cancer in acromegaly [3], [10]–[14], [16], [17], reflecting its greater frequency amongst the various DTC histotypes in the general population too. BRAF mutations have proved to be the most common genetic event involved in the onset of PTC in the general population, responsible for about 45% of cases; other frequently-identified genetic events include point mutations of the *RAS* genes and RET proto-oncogene chromosome rearrangements.

Mutations in the aryl-hydrocarbon receptor (AHR) interacting protein (AIP) and p27^KIP1^ encoding gene *CDKN1B* have recently been associated with familial forms of acromegaly [18]–[20], though no evidence has been produced to support a role for these genes in sporadic endocrine neoplasms other than pituitary adenoma [21], [22], or in the higher cancer risk seen in acromegalic patients [23]. AIP down-regulation in GH-secreting adenomas has also been associated with tumor aggressiveness [24], [25]. To our knowledge, AIP and AHR expression has not been studied in the thyroid or in thyroid DTC, and certainly not in acromegaly.

The aims of our study were: 1) to ascertain the prevalence of DTC in a large cohort of acromegalic patients followed up at our institutions; 2) to describe the clinical features and outcome of acromegaly associated with DTC; and 3) to assess the putative role of some known pro-oncogenic factors in acromegaly-related DTC.

## Materials and Methods

### Ethics statement

All studies were performed in accordance with the guidelines proposed in the Declaration of Helsinki: the local ethical committee approved our study protocol and all patients gave their written informed consent.

### Subjects

We studied 113 consecutive patients referred to our endocrinology units (88 in Padua, 25 in Udine). The sample consisted of 50 males and 63 females with a mean age of 56±14 years (range 29–90); their mean age when acromegaly was diagnosed was 45±13 years (range 24–85). As of January 2013, the patients had a mean follow-up of 133±104 months (range 7–396) since their acromegaly was diagnosed. All patients came from borderline iodine intake areas (urinary iodine concentration: 80 mcg/l) [26].

Acromegaly was managed according to current international criteria [1], [27]. It was diagnosed on the strength of: clinical features; an unsuppressed GH nadir below 0.4 µg/L in the oral glucose tolerance test (OGTT); and high IGF-1 levels for gender and age in the European population. The disease was considered “*active*” when randomly-tested serum GH levels were ≥1 µg/L, IGF-1 (normalized for sex and age) was elevated in patients with clinical symptoms of active acromegaly, and with a GH nadir after the OGTT ≥0.4 µg/L, while the disease was judged to be “*controlled*” when GH levels were <1 µg/L and IGF-1 values were normal [27].

Thyroid ultrasound (US) was performed at diagnosis, then every 12 months in patients with goiter, and every 3 years in patients with normal thyroid US findings, or promptly whenever there was clinical evidence of a thyroid nodule. Fine-needle aspiration biopsy (FNAB) was performed on single thyroid nodules ≥1 cm or thyroid nodules of any size with at least one suspect US feature (rapid growth, marked hypoechogenicity, irregular or microlobulated margins, microcalcifications, increased vascularization or inelastic tissue on elastosonography).

### Genetics

Germline mutations in the *AIP* gene were investigated in all patients, analyzing the whole coding region, intron-exon boundaries, 5′- and 3′-UTRs, and large rearrangements using Multiplex Ligation-dependent Probe Amplification, as described elsewhere [23].

A thyroid sample was available for molecular analysis in all PTC cases. After sections stained with hematoxylin and eosin had been examined, cancer tissue specimens containing more than 60–70% of tumor cells were chosen for molecular analysis. Genomic DNA was extracted from tissues using the DNeasy Blood and Tissue kit (Qiagen) according to the manufacturer's protocol. Analyses were performed for *BRAF* (NM_004333.4) (exon 15), N-*RAS* (NM_002524.3) (exons 2 and 3), K-*RAS* (NM_033360.2) (exons 2 and 3), and H-*RAS* (NM_005343.2) (exons 2 and 3) mutations by direct sequencing, as reported elsewhere [28].

### Immunohistochemistry for AIP and AHR

In the PTC cases collected for molecular analysis, immunohistochemistry was performed at the University of L'Aquila as described elsewhere (24), always using a mouse monoclonal anti-AIP antibody diluted 1∶500 (clone 35-2, Novus Biologicals LLC, Littleton, CO, USA, distributed by DBA Italia, Milan, Italy) and a polyclonal rabbit anti-AHR antibody diluted 1∶50 (sc-5579, Santa Cruz Biotechnology, Santa Cruz, CA, USA, distributed by DBA Italia, Milan, Italy). Normal pituitary samples were used as positive controls. AIP and AHR protein expression was also analyzed in a set of 6 specimens of PTC unrelated to acromegaly, 3 carrying a BRAF V600E mutation, and 3 wild-type for BRAF. For each case of PTC, normal thyroid tissue obtained adjacent to neoplastic areas was also analyzed. Slides were photographed using a Zeiss Axioplan 2 microscope (Carl Zeiss Microimaging Inc., USA) and a Leica DFC 320 digital camera (Leica GmbH, Germany).

Immunostaining for AIP and AHR was classified semi-quantitatively as follows: 0 if ≤30% of cells were stained; 1 for >30 and ≤60% of stained cells; 2 for >60 and ≤80% of stained cells; and 3 for >80% of stained cells. The intensity of staining was indicated as: weak, moderate or strong.

For AHR, the subcellular localization of staining (cytoplasmic, nuclear or both) was also considered.

### Statistical analysis

We calculated proportions and rates for categorical variables, means ± standard deviations, or medians and ranges for parametric or non-parametric variables. Groups were compared with the chi-square test for categorical variables (or Fisher's exact test when the cell count was <5), or with the Mann-Whitney test for quantitative variables, as appropriate. The SPSS 17 software package (SPSS, Inc., Chicago, IL) was used to manage the dataset and for the statistical analyses. The significance level was set at p<0.05 for all tests.

## Results

### Clinical examination: the onset of DTC does not depend on the activity and duration of acromegaly

We retrospectively studied the files on a consecutive series of 113 acromegalic patients whose clinical characteristics are listed in Table 1. The overall prevalence of goiter identified by US was 73% (83/113 patients): 27 had uninodular, 43 had multinodular and 13 had diffuse goiter. Among the 27 patients with uninodular goiter, the nodule's diameter was ≥1 cm in 55%, while in the other 45% it was <1 cm: in this latter group, 5 patients had at least one suspect US feature (2 manifested rapid growth, one marked hypoechogenicity and microcalcifications, 2 increased vascularization and inelastic tissue on elastosonography), so FNAB was performed. In 12 patients, the final histology after thyroid surgery revealed a DTC, 10 of which were PTCs and 2 were cases of follicular thyroid carcinoma (FTC). The overall prevalence of thyroid cancer in our study population was 11%.

**Table 1:** Clinical characteristics of acromegalic patients with and without differentiated thyroid carcinoma (DTC).

*Characteristic*	*With DTC (n = 12)*	*Without DTC (n = 101)*	*p*
Age at acromegaly diagnosis (years)	53±16	45±13	**<0.05**
Follow-up for acromegaly (months)	126±99	133±105	0.83
GH at acromegaly diagnosis (µg/L)	25±25	34±58	0.69
IGF-1 at acromegaly diagnosis (µg/L)	767±311	866±360	0.43
Female	11 (92%)	53 (52%)	**<0.01**
Pituitary macroadenoma	8 (67%)	80 (79%)	0.33
Pituitary neurosurgery	7 (59%)	78 (77%)	0.16
Pituitary radiotherapy	2 (17%)	23 (23%)	0.63
Goiter	12 (100%)	71 (70%)	**<0.05**
Uninodular goiter	8 (67%)	19 (27%)	**<0.01**
Multinodular goiter	4 (33%)	39 (55%)	**<0.01**
Diffuse goiter	0 (0%)	13 (18%)	**<0.01**
Other tumors	7 (58%)	51 (50%)	0.61

GH/IGF-1 levels at diagnosis, and the time since acromegaly was diagnosed did not differ between patients with and without DTC. Patients with DTC were older (53 versus 45 years, p<0.05) and mostly female (11 out of 12). There was a significant association between goiter and DTC (12 out of 12 cases): DTC patients mainly had uninodular goiter (67%), whereas the goiter in acromegalic patients without DTC tended more often to be multinodular (55%) or diffuse (18%).

DTC was diagnosed before acromegaly in 4 cases, during the initial diagnostic work-up for acromegaly in 1 patient, and during the follow-up for acromegaly in 6 (1 with controlled and 5 with active acromegaly), from 10 to 120 months after their GH excess had been diagnosed; in one case, DTC was diagnosed 31 years before the patient's acromegaly came to light so the two conditions were probably not related; and in one patient, the thyroid malignancy was detected during a period of GH deficiency (due to neurosurgery and radiotherapy). Table 2 shows the clinical characteristics of the 12 patients with DTC, who were all referred for routine follow-up for their thyroid cancer. All patients were treated with post-operative radio-iodine ablation therapy, and they were all considered “disease-free” after a median follow-up of 117 months (range 7–360).

**Table 2:** Clinical characteristics, histotypes, and follow-up for acromegaly and thyroid cancer in 12 acromegalic patients with DTC.

Acromegaly						DTC						
*n, sex, age*	*Diagnosis (yrs)*	*FU (months)*	*Pituitary adenoma*	*N Sur*	*RT*	*Diagnosis (yrs)*	FU (months)	IGF-1 µg/L (ULN)	TNM	BRAF V600E	AHR score	Status
1,F,66	37	360	macro	yes	yes	56	132	118 (0.48*)	PTC, pT1N0	wt	2	ADF
2,F,44	35	115	macro	yes	no	35	117	347 (1.41)	PTC, pT1N0	mut	3	ADF
3,F,76	63	162	macro	no	no	67	111	652 (1.74)	PTC, pT1a(m)NX	mut	3	ADF
4,F,73	67	72	macro	no	no	66	75	695 (3.44)	PTC, pT2(m)N1a	mut	3	ADF
5,M,60	58	31	macro	no	no	27	396	n.a.	FTC, pT2N1	n.a.	n.a.	ADF
6,F,86	85	21	macro	no	no	77	109	n.a.	PTC, pT3b NX	wt	3	ADF
7,F,68	68	7	micro	no	no	68	7	520 (2.57)	PTC, pT3N1b	mut	3	ADF
8,F,62	53	120	macro	yes	no	53	120	640 (2.59)	PTC, pT1(m)NX	mut	3	ADF
9,F,58	30	190	macro	yes	yes	45	145	320 (1.30)	PTC, pT1NX	mut	3	ADF
10,F,68	51	180	micro	yes	no	70	170	120 (0.49)	PTC, pT1N0	wt	2	ADF
11,F,45	39	70	micro	yes	no	37	75	n.a.	PTC, pT1a,NX	mut	3	ADF
12,F,68	45	190	micro	yes	no	63	64	380 (1.88)	FTC, pT1N0	n.a.	n.a.	ADF

Legend: FU: follow-up, N Sur: neurosurgery; RT: radiotherapy, DTC: differentiated thyroid carcinoma, PTC: papillary thyroid carcinoma, FTC: follicular thyroid carcinoma;

*: DTC diagnosed during episode of hypopituitarism; ULN: upper limit of normality for sex and age; ADF: alive, disease-free. T1a: nodes with largest diameter ≤10 mm; (m) multifocal; mut: BRAF V600E mutation; wt: wild-type; n.a.: not applicable.

### AIP, BRAF and H-K-N-RAS status in acromegalic patients

The classical BRAF-exon 15 V600E point mutation was found in 70% of PTC specimens. No N-*RAS*, H-*RAS* or K-*RAS* gene mutations were identified. Two germline *AIP* changes came to light among the acromegalic patients with DTC: one was a c.911G>A (R304Q) variant, which had previously been reported and considered a variant of uncertain significance [23], [29]; the other was a synonymous substitution c.144G>A (T48T), considered a rare neutral polymorphism [23].

### Immunohistochemistry for AIP and AHR: PTCs revealed a high AHR protein expression irrespective of patients' acromegaly status

**Figure 1:**
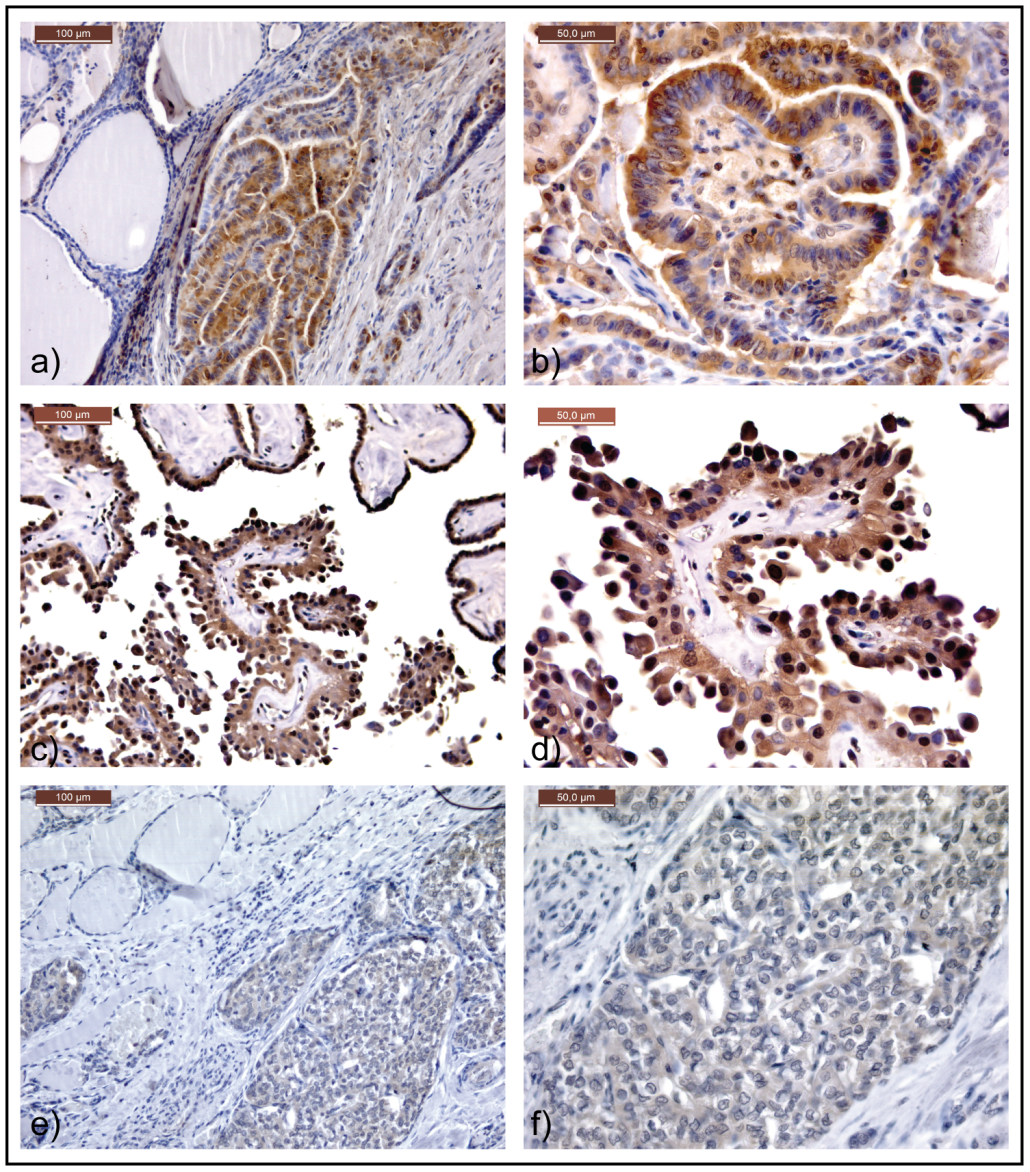
AHR protein expression in: an acromegaly-related classical variant of PTC carrying a V600E BRAF mutation (a–b); an acromegaly-unrelated PTC variant with hobnail features carrying a V600E BRAF mutation (c–d); an acromegaly-unrelated follicular variant of PTC, wild-type for BRAF (e–f). Normal thyroid tissue surrounding cancer cells is also visible (a–e). a, c, e: original magnification x20; b, d, f: original magnification x40.

PTCs, in patients with and without acromegaly, revealed little or no AIP immunostaining, and no differences vis-à-vis the paired normal thyroid tissues. A similarly weak/absent cytoplasmic and nuclear immunostaining was observed for AHR in normal thyroid areas adjacent to neoplastic tissues. On the other hand, all PTCs (in patients with and without acromegaly) showed quite extensive staining, with percentages of stained cells scored as “2” or “3” in all cases (see Table 2 and Table S1). Immunostaining for AHR in PTC cells was detected consistently in the cytoplasm, while nuclear immunostaining was more heterogeneous (figure 1). The strongest AHR staining, in terms of intensity and percentage of positive cells, was seen in PTC samples carrying a BRAF V600E mutation (all cases scored “3” for the percentage of stained cells): the median score for AHR staining was “2” for BRAF-wild type PTC and “3” for BRAF-mutated PTC (p<0.01). There was no any difference in AHR staining score between PTCs in patients with versus without acromegaly.

## Discussion

Several multicenter studies have reported a higher risk of benign and malignant tumors developing in acromegalic patients compared to the general population, often involving the digestive tract (and the colorectum in particular), breast, brain, lung, uterus, adrenal gland and prostate [1]–[4]. Cancer-related mortality ranged from 15% to 24% in these studies. Differentiated thyroid carcinomas are also among the malignancies most frequently reported in association with acromegaly [17], and benign thyroid conditions such as diffuse and multinodular goiter are also common in this setting.

In a cohort of 113 acromegalic patients, we found a 73% prevalence of diffuse and nodular goiter, which is similar to figures reported elsewhere in the literature (Table 3). The prevalence of DTC in our series was 11%, slightly higher than previously reported, and much higher than the estimated 0.1% in the general population in mildly iodine-deficient areas [12]. Analyzing data in the literature, the rate of DTC in acromegaly varies considerably, ranging from 1% to 11%, and averaging around 5% overall (Table 3); this figure was calculated without considering the national disease registry-based study by Orme et al., which may have underestimated thyroid disease. We noted a gradual increase in the number of DTC cases reported from the earliest to the latest studies, which probably simply reflects the higher DTC incidence rate observed in the last decades [30], [31] thanks to better diagnostic procedures, and to the use of US and FNAB in particular. The higher DTC prevalence seen in our series is likewise attributable to improved screening methods [31]. Certainly all of our patients were closely followed up with US, as in the study by Dagdelen S et al. [17], which reported a very similar prevalence of DTC (10.8%). Our series of patients had quite a long follow-up (mean 11 years) by comparison with previous studies, and this might also partially account for our high DTC detection rate. Although there was no gender preponderance in our cohort of acromegalic patients, the vast majority of those with DTC were female (11 out of 12), reflecting a trend seen in the general population.

**Table 3:** Publications on the prevalence of DTC in acromegaly.

Reference	Patients n	M/F (%)	Prevalence of goiter (%)	Prevalence of DTC (%) (n PTC/n FTC)	M/F with DTC	DTC follow-up (months)
Popovic V *et al*., 1998 [10]	220	38%/62%	n.a.	1.4% (3 PTC/0 FTC)	n.a.	n.a.
Gasperi M *et al*., 2002 [11]	258	43%/57%	72%	1.2% (3 PTC/0 FTC)	n.a.	n.a.
Herrmann BL *et al.*, 2004 [12]	73	n.a.	82%	5.5% (3 PTC/1 FTC)	n.a.	n.a.
Tita P *et al.*, 2005 [13]	125	44%/56%	82%	5.6% (5 PTC/2 FTC)	4/3	6–140
Kurimoto M *et al.*, 2008 [3]	140	39%/61%	57%	3.6% (4 PTC/1 n.a)	1/3	n.a.
Gullu BE *et al.*, 2010 [14]	105	38%/62%	62%	4.8% (5 PTC/0 FTC)	5/0	n.a.
Baldys-Waligorska A *et al.*, 2010 [15]	101	30%/70%	63%	3% (1 PTC/2 FTC)	n.a.	n.a.
Dos Santos MCC *et al.*, 2013 [16]	124	39%/61%	74%	7.3% (9 PTC/0 FTC)	3/6	n.a.
Dagdelen S *et al*., 2013 [17]	160	51%/49%	80%	10.6% (13 PTC/3 FTC/1 PTC-FTC)	8/9	n.a.
*Present paper*	*113*	*44%/56%*	*73%*	*10.6% (10 PTC/2 FTC)*	*1/11*	7–396
**Overall**	**1419**	**41%/59%**	**72%**	4.8% (57 PTC/10 FTC)*	**14/23**	

Legend: PTC: papillary thyroid carcinoma, FTC: follicular thyroid carcinoma; M: Male; F: Female.

Several mechanisms have been suggested to explain the onset of DTC in acromegaly. In recent years, experimental evidence has led to the suggestion that there is an autocrine/paracrine loop for the IGF system in human PTC carcinogenesis [32]–[34]. In acromegaly, higher serum GH and IGF-1 levels probably cooperate synergically with such a loop in promoting thyrocyte proliferation and transformation. The relationship between the onset of DTC and the duration/severity of acromegaly is rather controversial, however. As in previous reports [9]–[17], we found no correlation between DTC risk and GH/IGF-1 levels or duration of acromegaly. So other factors must be involved in the pathogenesis of DTC in acromegalic patients, in addition to GH/IGF-1 excess, such as genetic or epigenetic modifications, like those already identified in the setting of PTC and GH-secreting pituitary adenoma. With this in mind, we looked at whether the main genetic drivers of DTC (BRAF and RAS mutations) were important in acromegaly-related DTC too, and we assessed the expression in DTC of the key molecular drivers of acromegaly (AIP and AHR).

To our knowledge, this is the first study to analyze the prevalence of BRAF/RAS mutations in acromegaly-related DTC. In our sample, the most common oncotype in acromegaly-related DTC was PTC (10 out of 12 cases; see Table 3) with a high frequency (70%) of BRAF V600E mutations, whereas none of our cases harbored RAS mutations. Although the limited number of PTCs in the present series prevents us from drawing any final conclusions on the prevalence of BRAF mutations in PTCs in patients with versus without acromegaly, BRAF was the main genetic driver of follicular cell transformation in our cases of acromegaly-related PTC. It is worth adding that all our patients with DTC (including the BRAF-positive PTC patients) had a good prognosis and were judged “disease-free” at the end of the follow-up.

Germline mutations in the *AIP* gene have been associated with a predisposition to GH-secreting pituitary adenoma [19], [24]. Our cohort of patients had no confirmed germinal *AIP* mutations, but a PTC was diagnosed in one patient with an AIP R304Q variant and a concomitant somatic BRAF V600E mutation. AHR is a cytosolic ligand-activated transcription factor that exists in an inactive state in the absence of the ligand, as part of the cytosolic multimeric protein complex in which AIP is one of the partners. AHR has been studied for its capacity to bind environmental xenobiotics: this interaction activates AHR, which then translocates into the nucleus, regulating the expression of genes encoding for cytochrome P450 enzymes and metabolizing exogenous ligands [35]. Apart from its function in regulating xenobiotic detoxification, AHR is attributed important roles in animal and human carcinogenesis too, through its effects on cell cycle regulation, apoptosis, extracellular matrix remodeling, and angiogenesis. Recent works have shown that, even without its environmental ligands, AHR is overexpressed and constitutively active in a variety of sporadic human cancers, especially mammary, prostate, gastric and lung cancers [35]. AIP and AHR proteins are both expressed in normal pituitary tissue, and our data are the first to show that, while they are poorly expressed in the normal thyroid, AHR is selectively overexpressed in acromegaly-related PTC. The increased AHR expression was consistently seen in the cytoplasm of PTC cells, while the picture was less homogeneous in the nucleus. The molecular mechanisms involved in AHR overexpression in PTC are still not known. We found that PTC samples harboring a BRAF V600E mutation had a significantly more marked increase in AHR protein expression. This prompted us to examine a series of PTC specimens from patients without acromegaly too, to see if this finding was unique to acromegalic patients or common to all PTCs. Unexpectedly, PTCs unassociated with acromegaly revealed a high AHR protein expression as well, which was particularly evident in the BRAF-mutated cases. This AHR overexpression in PTC (particularly in BRAF-mutated cases) irrespective of a patient's acromegaly status could reflect a close relationship between AHR and BRAF. It has been demonstrated that TCCD (2,3,7,8,-tetrachlorodibenzo-p-dioxin) and other AHR xenobiotic ligands induce ERK1/2 phosphorylation [36], as observed in the presence of BRAF mutations [37]. It has also been demonstrated that a preserved MAPK pathway activity is essential for AHR to function as a transcription factor and induce AHR-regulated genes [36]. Whether AHR activation is merely an epiphenomenon of MAPK pathway activation by mutated BRAF, or involved in the mechanisms behind PTC pathogenesis remains to be seen, however, and our data need to be confirmed in larger series of PTCs with and without BRAF mutations.

In conclusion, we confirmed that acromegalic patients carry a risk of DTC (up to 11% higher than in the general population, in our experience), and clinicians should periodically check their patients' thyroid morphology using US, FNAB cytology, and even molecular analysis, as necessary, in accordance with the current guidelines of the major societies on thyroid diseases [38]. In the last few years, several consensus documents have been published on the management of acromegaly-related complications, but even the most recent do not mention any long-term clinical and US thyroid assessment for these patients [39]. We suggest bearing thyroid cancer in mind during the follow-up of acromegalic patients because it is not uncommon. PTC in acromegaly shares some of the molecular events occurring in other PTC patients, such as somatic BRAF mutations. The acromegaly-related genetic background alone (notably a systemic or local GH/IGF-1 overproduction) is probably not enough to trigger the onset of follicular epithelial cell-derived cancer. Judging from our preliminary findings, it would now seem reasonable to postulate an integrated model in which acromegaly would facilitate the acquisition of the somatic genetic drivers responsible for PTC and, for the first time, we hypothesize an original, hitherto unknown role for AHR in PTC.

## Supporting Information

Table S1 **Clinical characteristics, histotypes, BRAF status and AHR score in PTC in patients without acromegaly.** Legend: mut: BRAF V600E mutation; wt: wild-type.
(DOC)
