# Supplementary material for: AHR Over-Expression in Papillary Thyroid Carcinoma: Clinical and Molecular Assessments in a Series of Italian Acromegalic Patients with a Long-Term Follow-Up
Source: PLoS One. 2014 Jul 14;9(7):e101560. doi: 10.1371/journal.pone.0101560 (PMC4096503; doi:10.1371/journal.pone.0101560)
Supplement: Table S1 — Clinical characteristics, histotypes, BRAF status and AHR score in PTC in patients without acromegaly. Legend: mut: BRAF V600E mutation; wt: wild-type. (DOC) [file pone.0101560.s001.doc]

***Table S1:*** *Clinical characteristics, histotypes, BRAF status and AHR score in PTC in patients without acromegaly.*

| *n, sex, age* | *Diagnosis (yrs)* | *TNM* | *BRAF V600E* | *AHR score* |
| --- | --- | --- | --- | --- |
| 1,F,70 | 68 | PTC, pT3N0 | mut | 3 |
| 2,F,77 | 75 | PTC, pT3NX | mut | 3 |
| 3,F,50 | 48 | PTC, pT1N0 | wt | 2 |
| 4,F,38 | 37 | PTC, pT3N0 | wt | 2 |
| 5,F,60 | 59 | PTC, pT1N0 | wt | 2 |
| 6,F,62 | 62 | PTC, pT2N0 | mut | 3 |

Legend: mut: BRAF V600E mutation; wt: wild-type.
